# Bis{(*E*)-2-[1-(eth­oxy­imino)­eth­yl]-1-naphtho­lato-κ^2^ *N*,*O* ^1^}copper(II)

**DOI:** 10.1107/S1600536810047574

**Published:** 2010-11-24

**Authors:** Wen-Kui Dong, Xiu-Yan Dong, Yin-Xia Sun, Jian-Chao Wu, Si-Jia Xing

**Affiliations:** a School of Chemical and Biological Engineering, Lanzhou Jiaotong University, Lanzhou 730070, People’s Republic of China

## Abstract

In the title complex, [Cu(C_14_H_14_NO_2_)_2_], the discrete complex mol­ecules have crystallographic inversion symmetry. The slightly distorted square-planar coordination sphere of the Cu^II^ atom comprises two phenolate O atoms and two oxime N atoms from two bidentate–chelate 2-[1-(eth­oxy­imino)­eth­yl]-1-naphtho­late *O*-ethyl oxime (*L* ^−^) ligands [Cu—O = 1.8919 (17) Å and Cu—N = 1.988 (2) Å]. The two naphthalene ring systems in the mol­ecule are parallel, with a perpendicular inter­planar spacing of 1.473 (2) Å, while each complex unit forms links to four other mol­ecules *via* inter­molecular methyl C—H⋯π inter­actions, giving an infinite cross-linked layered supra­molecular structure

## Related literature

For background to oximes, see: Chaudhuri (2003 ▶); Dong *et al.* (2007 ▶, 2008 ▶). For related structures, see: Zhao *et al.* (2009 ▶); Dong, Zhao *et al.* (2009 ▶). For the synthesis of the title complex, see: Dong, Tong *et al.* (2009 ▶). For the biological activity of copper(II) complexes, see: Karmaka *et al.* (2007 ▶).
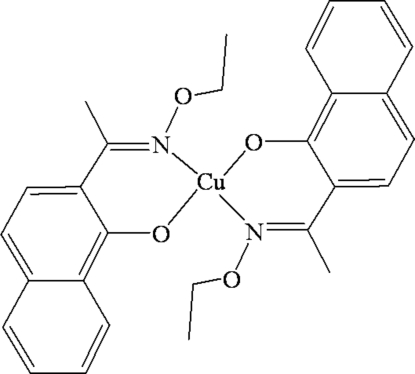


## Experimental

### 

#### Crystal data

[Cu(C_14_H_14_NO_2_)_2_]
*M* *_r_* = 520.06
Monoclinic, 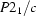

*a* = 11.317 (1) Å
*b* = 7.1092 (8) Å
*c* = 15.171 (2) Å
β = 96.317 (1)°
*V* = 1213.1 (2) Å^3^
*Z* = 2
Mo *K*α radiation
μ = 0.94 mm^−1^
*T* = 298 K
0.17 × 0.15 × 0.10 mm

#### Data collection

Bruker SMART CCD area-detector diffractometer
Absorption correction: multi-scan (*SADABS*; Sheldrick, 1996 ▶) *T*_min_ = 0.857, *T*_max_ = 0.912
6095 measured reflections
2130 independent reflections
1490 reflections with *I* > 2σ(*I*)
*R*_int_ = 0.044

#### Refinement

*R*[*F* ^2^ > 2σ(*F* ^2^)] = 0.036
*wR*(*F* ^2^) = 0.081
*S* = 1.01
2130 reflections
162 parameters
H-atom parameters constrained
Δρ_max_ = 0.22 e Å^−3^
Δρ_min_ = −0.21 e Å^−3^

### 

Data collection: *SMART* (Siemens, 1996 ▶); cell refinement: *SAINT* (Siemens, 1996 ▶); data reduction: *SAINT*; program(s) used to solve structure: *SHELXS97* (Sheldrick, 2008 ▶); program(s) used to refine structure: *SHELXL97* (Sheldrick, 2008 ▶); molecular graphics: *SHELXTL* (Sheldrick, 2008 ▶); software used to prepare material for publication: *SHELXTL*.

## Supplementary Material

Crystal structure: contains datablocks global, I. DOI: 10.1107/S1600536810047574/zs2078sup1.cif

Structure factors: contains datablocks I. DOI: 10.1107/S1600536810047574/zs2078Isup2.hkl

Additional supplementary materials:  crystallographic information; 3D view; checkCIF report

## Figures and Tables

**Table 1:** Hydrogen-bond geometry (Å, °) *Cg*1 is the centroid of the C9–C14 ring.

*D*—H⋯*A*	*D*—H	H⋯*A*	*D*⋯*A*	*D*—H⋯*A*
C3—H3*A*⋯*Cg*1^i^	0.96	2.66	3.530 (3)	151

Symmetry code: (i) 
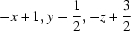
.

**Fig. 1.**
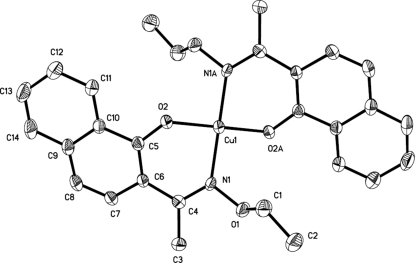
The molecule structure of the title complex with the atom numbering scheme [Symmetry code: (A) -x + 1, -y + 1, -z + 1]. Displacement ellipsoids for non-hydrogen atoms are drawn at the 30% probability level.

**Fig. 2.**
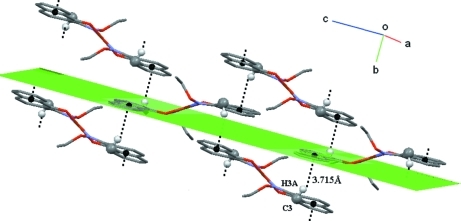
Part of the supramolecular structure of the title complex with C—H···π interactions shown as dashed lines.

## supplementary crystallographic information

### Comment

Oxime-type compounds are a versatile class of organic ligands widely used in coordination and analytical chemistry and extraction metallurgy (Dong *et al.*, 2007; Dong *et al.*, 2008; Chaudhuri, 2003). Due to their chelating ability and positive redox potential, many copper(II) complexes are generally biologically active (Karmaka *et al.*, 2007). As part of our ongoing research into the transition metal complexes with oxime-type ligands (Dong, Tong *et al.*, 2009), we report here the synthesis and crystal structures of the title Cu^II^ complex with 1-(1-hydroxynapthalen-2-yl)ethanone *O*-ethyl oxime (HL), the title compound [Cu(C_14_H_14_NO_2_)_2_] (I) (Fig. 1).

In the crystal structure of (I) the discrete complex molecules have inversion symmetry, the slightly distorted square-planar four-coordinate *trans*-CuN_2_O_2_ coordination sphere comprising two phenolic O-atoms and two oxime N-atoms from two bidentate-chelate *L*^-^ ligands [Cu(1)—O(2), 1.8919 (17) Å; Cu(1)—N(1), 1.988 (2) Å]. These bond distances are within the normal range observed in a similar Cu^II^ complex (Dong, Zhao *et al.*, 2009). The two naphthalene rings of the ligands in the complex molecule are parallel with a perpendicular interplanar spacing of 1.473 (2) Å. In the crystal structure, the complex molecules are linked by intermolecular methyl C—H···π interactions involving the naphthalene ring C5—C14, with a C3—H3A···π ring centroid separation of 3.715 (2) Å. Thus, every complex molecule forms links with four other adjacent molecules giving an infinite supramolecular layer structure (Fig. 2).

### Experimental

1-(1-Hydroxynapthalen-2-yl)ethanone *O*-ethyl oxime (HL) was synthesized using a method similar to one reported previously (Zhao *et al.*, 2009). Yield, 62.9%. m.p. 315–316 K. Anal. Calcd for C_14_H_15_NO_2_: C, 73.34; H, 6.59; N,6.11%. Found: C, 73.30; H, 6.52; N, 6.22%. A solution of Cu^II^ acetate monohydrate (2.5 mg, 0.012 mmol) in methanol (3 ml) was added dropwise to a solution of HL (5.6 mg, 0.023 mmol) and 99% triethylamine (0.025 ml) in methanol (3 ml) at room temperature. The color of the mixing solution turned to yellow immediately, then turned to brown slowly after which the filtrate was allowed to stand at room temperature for about two weeks. The solvent was partially evaporated and brown single crystals suitable for X-ray crystallographic analysis were obtained. Anal. Calcd. for [Cu(*L*)_2_] (C_28_H_28_CuN_2_O_4_): C, 64.66; H, 5.43; N, 5.39; Cu, 12.22%. Found: C, 64.70; H, 5.49; N, 5.33; Cu, 12.20%.

### Refinement

H atoms were placed in calculated positions and non-H atoms were refined anisotropically. H atoms were treated as riding atoms with distances C—H = 0.96 Å (CH_3_), C—H = 0.97 Å (CH_2_) and 0.93 Å (CH). The isotropic displacement parameters for all H atoms were set equal to 1.2 or 1.5 *U*_eq_ of the carrier atom.

### Crystal data

**Table:**

[Cu(C_14_H_14_NO_2_)_2_]	*F*(000) = 542
*M**_r_* = 520.06	*D*_x_ = 1.424 Mg m^−^^3^
Monoclinic, *P*2_1_/*c*	Melting point = 315–316 K
Hall symbol: -P 2ybc	Mo *K*α radiation, λ = 0.71073 Å
*a* = 11.317 (1) Å	Cell parameters from 1730 reflections
*b* = 7.1092 (8) Å	θ = 2.7–25.5°
*c* = 15.171 (2) Å	µ = 0.94 mm^−^^1^
β = 96.317 (1)°	*T* = 298 K
*V* = 1213.1 (2) Å^3^	Block-like, brown
*Z* = 2	0.17 × 0.15 × 0.10 mm

### Data collection

**Table:**

Bruker SMART CCD area-detector diffractometer	2130 independent reflections
Radiation source: fine-focus sealed tube	1490 reflections with *I* > 2σ(*I*)
graphite	*R*_int_ = 0.044
φ and ω scans	θ_max_ = 25.0°, θ_min_ = 1.8°
Absorption correction: multi-scan (*SADABS*; Sheldrick, 1996)	*h* = −10→13
*T*_min_ = 0.857, *T*_max_ = 0.912	*k* = −8→8
6095 measured reflections	*l* = −18→18

### Refinement

**Table:**

Refinement on *F*^2^	Primary atom site location: structure-invariant direct methods
Least-squares matrix: full	Secondary atom site location: difference Fourier map
*R*[*F*^2^ > 2σ(*F*^2^)] = 0.036	Hydrogen site location: inferred from neighbouring sites
*wR*(*F*^2^) = 0.081	H-atom parameters constrained
*S* = 1.01	*w* = 1/[σ^2^(*F*_o_^2^) + (0.0342*P*)^2^] where *P* = (*F*_o_^2^ + 2*F*_c_^2^)/3
2130 reflections	(Δ/σ)_max_ < 0.001
162 parameters	Δρ_max_ = 0.22 e Å^−^^3^
0 restraints	Δρ_min_ = −0.20 e Å^−^^3^

### Special details

**Table:**

Geometry. All e.s.d.'s (except the e.s.d. in the dihedral angle between two l.s. planes) are estimated using the full covariance matrix. The cell e.s.d.'s are taken into account individually in the estimation of e.s.d.'s in distances, angles and torsion angles; correlations between e.s.d.'s in cell parameters are only used when they are defined by crystal symmetry. An approximate (isotropic) treatment of cell e.s.d.'s is used for estimating e.s.d.'s involving l.s. planes.
Refinement. Refinement of *F*^2^ against ALL reflections. The weighted *R*-factor *wR* and goodness of fit *S* are based on *F*^2^, conventional *R*-factors *R* are based on *F*, with *F* set to zero for negative *F*^2^. The threshold expression of *F*^2^ > σ(*F*^2^) is used only for calculating *R*-factors(gt) *etc*. and is not relevant to the choice of reflections for refinement. *R*-factors based on *F*^2^ are statistically about twice as large as those based on *F*, and *R*- factors based on ALL data will be even larger.

### Fractional atomic coordinates and isotropic or equivalent isotropic displacement parameters (Å^2^)

**Table:**

	*x*	*y*	*z*	*U*_iso_*/*U*_eq_	
Cu1	0.5000	0.5000	0.5000	0.03833 (17)	
N1	0.63479 (18)	0.5872 (3)	0.58534 (14)	0.0381 (6)	
O1	0.75134 (16)	0.6050 (3)	0.55763 (12)	0.0514 (6)	
O2	0.39686 (15)	0.6209 (3)	0.57215 (12)	0.0462 (5)	
C1	0.7502 (3)	0.7498 (5)	0.49108 (19)	0.0576 (9)	
H1A	0.7131	0.8627	0.5111	0.069*	
H1B	0.7052	0.7081	0.4365	0.069*	
C2	0.8761 (3)	0.7901 (6)	0.4756 (2)	0.0789 (12)	
H2A	0.9179	0.8426	0.5284	0.118*	
H2B	0.8770	0.8781	0.4277	0.118*	
H2C	0.9141	0.6754	0.4608	0.118*	
C3	0.7501 (2)	0.6552 (4)	0.72791 (18)	0.0479 (8)	
H3A	0.7842	0.5401	0.7521	0.072*	
H3B	0.7344	0.7376	0.7754	0.072*	
H3C	0.8047	0.7149	0.6926	0.072*	
C4	0.6356 (2)	0.6131 (4)	0.67096 (17)	0.0359 (7)	
C5	0.4124 (2)	0.6230 (4)	0.65963 (17)	0.0371 (7)	
C6	0.5233 (2)	0.6069 (4)	0.71082 (17)	0.0343 (6)	
C7	0.5266 (3)	0.5992 (4)	0.80539 (17)	0.0416 (7)	
H7	0.5994	0.5806	0.8391	0.050*	
C8	0.4279 (3)	0.6180 (4)	0.84775 (19)	0.0468 (8)	
H8	0.4345	0.6127	0.9093	0.056*	
C9	0.3147 (3)	0.6458 (4)	0.79925 (19)	0.0436 (7)	
C10	0.3059 (2)	0.6458 (4)	0.70468 (18)	0.0397 (7)	
C11	0.1938 (3)	0.6651 (4)	0.6560 (2)	0.0525 (9)	
H11	0.1878	0.6652	0.5944	0.063*	
C12	0.0930 (3)	0.6838 (5)	0.6976 (2)	0.0690 (11)	
H12	0.0193	0.6941	0.6642	0.083*	
C13	0.1009 (3)	0.6873 (5)	0.7907 (3)	0.0701 (11)	
H13	0.0324	0.7016	0.8188	0.084*	
C14	0.2079 (3)	0.6699 (4)	0.8396 (2)	0.0597 (9)	
H14	0.2117	0.6738	0.9012	0.072*	

### Atomic displacement parameters (Å^2^)

**Table:**

	*U*^11^	*U*^22^	*U*^33^	*U*^12^	*U*^13^	*U*^23^
Cu1	0.0347 (3)	0.0528 (3)	0.0283 (3)	−0.0002 (3)	0.00671 (19)	−0.0033 (3)
N1	0.0276 (12)	0.0548 (15)	0.0336 (13)	0.0001 (11)	0.0112 (10)	0.0019 (11)
O1	0.0376 (11)	0.0795 (16)	0.0378 (12)	−0.0024 (11)	0.0064 (9)	0.0103 (11)
O2	0.0370 (11)	0.0722 (15)	0.0297 (11)	0.0050 (10)	0.0047 (9)	−0.0089 (10)
C1	0.057 (2)	0.068 (2)	0.049 (2)	−0.0095 (18)	0.0098 (16)	0.0089 (18)
C2	0.063 (2)	0.115 (3)	0.062 (2)	−0.026 (2)	0.0169 (18)	0.010 (2)
C3	0.0407 (17)	0.061 (2)	0.0410 (17)	−0.0053 (15)	0.0005 (14)	−0.0029 (15)
C4	0.0426 (17)	0.0317 (17)	0.0331 (16)	−0.0011 (13)	0.0028 (13)	0.0029 (13)
C5	0.0412 (17)	0.0341 (17)	0.0373 (17)	−0.0041 (13)	0.0106 (13)	−0.0056 (13)
C6	0.0407 (16)	0.0330 (16)	0.0304 (15)	−0.0024 (13)	0.0089 (13)	−0.0023 (13)
C7	0.0496 (18)	0.0411 (18)	0.0343 (16)	−0.0036 (15)	0.0058 (14)	−0.0022 (14)
C8	0.067 (2)	0.044 (2)	0.0312 (16)	−0.0051 (16)	0.0149 (15)	−0.0029 (14)
C9	0.0541 (19)	0.0359 (18)	0.0441 (18)	−0.0057 (14)	0.0203 (15)	−0.0037 (14)
C10	0.0414 (17)	0.0389 (18)	0.0407 (17)	−0.0043 (13)	0.0133 (14)	−0.0066 (13)
C11	0.0437 (19)	0.070 (2)	0.0452 (19)	0.0008 (16)	0.0136 (15)	−0.0055 (16)
C12	0.047 (2)	0.095 (3)	0.067 (3)	0.0030 (19)	0.0158 (18)	−0.005 (2)
C13	0.052 (2)	0.088 (3)	0.076 (3)	0.001 (2)	0.034 (2)	−0.003 (2)
C14	0.077 (2)	0.058 (2)	0.050 (2)	−0.0055 (19)	0.035 (2)	−0.0022 (17)

### Geometric parameters (Å, °)

**Table:**

Cu1—O2	1.8919 (17)	C4—C6	1.467 (3)
Cu1—O2^i^	1.8919 (17)	C5—C6	1.406 (4)
Cu1—N1	1.988 (2)	C5—C10	1.459 (3)
Cu1—N1^i^	1.988 (2)	C6—C7	1.432 (3)
N1—C4	1.311 (3)	C7—C8	1.355 (3)
N1—O1	1.433 (2)	C7—H7	0.9300
O1—C1	1.441 (3)	C8—C9	1.419 (4)
O2—C5	1.320 (3)	C8—H8	0.9300
C1—C2	1.497 (4)	C9—C14	1.425 (4)
C1—H1A	0.9700	C9—C10	1.427 (4)
C1—H1B	0.9700	C10—C11	1.403 (4)
C2—H2A	0.9600	C11—C12	1.369 (4)
C2—H2B	0.9600	C11—H11	0.9300
C2—H2C	0.9600	C12—C13	1.406 (4)
C3—C4	1.506 (3)	C12—H12	0.9300
C3—H3A	0.9600	C13—C14	1.355 (4)
C3—H3B	0.9600	C13—H13	0.9300
C3—H3C	0.9600	C14—H14	0.9300
			
O2—Cu1—O2^i^	180.00 (8)	C6—C4—C3	119.9 (2)
O2—Cu1—N1	87.68 (8)	O2—C5—C6	124.6 (2)
O2^i^—Cu1—N1	92.32 (8)	O2—C5—C10	116.5 (2)
O2—Cu1—N1^i^	92.32 (8)	C6—C5—C10	118.9 (2)
O2^i^—Cu1—N1^i^	87.68 (8)	C5—C6—C7	118.7 (2)
N1—Cu1—N1^i^	180.0	C5—C6—C4	122.1 (2)
C4—N1—O1	111.8 (2)	C7—C6—C4	119.1 (2)
C4—N1—Cu1	127.60 (18)	C8—C7—C6	122.6 (3)
O1—N1—Cu1	120.09 (14)	C8—C7—H7	118.7
N1—O1—C1	109.3 (2)	C6—C7—H7	118.7
C5—O2—Cu1	124.36 (17)	C7—C8—C9	120.8 (3)
O1—C1—C2	108.1 (3)	C7—C8—H8	119.6
O1—C1—H1A	110.1	C9—C8—H8	119.6
C2—C1—H1A	110.1	C8—C9—C14	123.7 (3)
O1—C1—H1B	110.1	C8—C9—C10	118.7 (2)
C2—C1—H1B	110.1	C14—C9—C10	117.6 (3)
H1A—C1—H1B	108.4	C11—C10—C9	119.2 (3)
C1—C2—H2A	109.5	C11—C10—C5	120.7 (2)
C1—C2—H2B	109.5	C9—C10—C5	120.1 (3)
H2A—C2—H2B	109.5	C12—C11—C10	121.2 (3)
C1—C2—H2C	109.5	C12—C11—H11	119.4
H2A—C2—H2C	109.5	C10—C11—H11	119.4
H2B—C2—H2C	109.5	C11—C12—C13	120.1 (3)
C4—C3—H3A	109.5	C11—C12—H12	120.0
C4—C3—H3B	109.5	C13—C12—H12	120.0
H3A—C3—H3B	109.5	C14—C13—C12	120.2 (3)
C4—C3—H3C	109.5	C14—C13—H13	119.9
H3A—C3—H3C	109.5	C12—C13—H13	119.9
H3B—C3—H3C	109.5	C13—C14—C9	121.7 (3)
N1—C4—C6	119.5 (2)	C13—C14—H14	119.2
N1—C4—C3	120.5 (2)	C9—C14—H14	119.2
			
O2—Cu1—N1—C4	31.5 (2)	C3—C4—C6—C7	−13.8 (4)
O2^i^—Cu1—N1—C4	−148.5 (2)	C5—C6—C7—C8	−4.0 (4)
O2—Cu1—N1—O1	−157.06 (19)	C4—C6—C7—C8	171.1 (3)
O2^i^—Cu1—N1—O1	22.94 (19)	C6—C7—C8—C9	0.3 (5)
C4—N1—O1—C1	−122.4 (3)	C7—C8—C9—C14	−178.8 (3)
Cu1—N1—O1—C1	64.9 (3)	C7—C8—C9—C10	2.6 (4)
N1—Cu1—O2—C5	−38.7 (2)	C8—C9—C10—C11	177.3 (3)
N1^i^—Cu1—O2—C5	141.3 (2)	C14—C9—C10—C11	−1.4 (4)
N1—O1—C1—C2	169.7 (2)	C8—C9—C10—C5	−1.7 (4)
O1—N1—C4—C6	177.8 (2)	C14—C9—C10—C5	179.6 (3)
Cu1—N1—C4—C6	−10.2 (4)	O2—C5—C10—C11	−0.3 (4)
O1—N1—C4—C3	−0.1 (4)	C6—C5—C10—C11	179.0 (3)
Cu1—N1—C4—C3	171.9 (2)	O2—C5—C10—C9	178.8 (2)
Cu1—O2—C5—C6	26.8 (4)	C6—C5—C10—C9	−1.9 (4)
Cu1—O2—C5—C10	−153.96 (19)	C9—C10—C11—C12	0.0 (5)
O2—C5—C6—C7	−176.0 (3)	C5—C10—C11—C12	179.0 (3)
C10—C5—C6—C7	4.7 (4)	C10—C11—C12—C13	1.2 (5)
O2—C5—C6—C4	8.9 (4)	C11—C12—C13—C14	−0.8 (6)
C10—C5—C6—C4	−170.3 (2)	C12—C13—C14—C9	−0.7 (5)
N1—C4—C6—C5	−16.8 (4)	C8—C9—C14—C13	−176.9 (3)
C3—C4—C6—C5	161.2 (3)	C10—C9—C14—C13	1.7 (5)
N1—C4—C6—C7	168.2 (3)		

Symmetry codes: (i) −*x*+1, −*y*+1, −*z*+1.

### Hydrogen-bond geometry (Å, °)

**Table:**

Cg1 is the centroid of the C9–C14 ring.

**Table:**

*D*—H···*A*	*D*—H	H···*A*	*D*···*A*	*D*—H···*A*
C3—H3A···Cg1^ii^	0.96	2.66	3.530 (3)	151

Symmetry codes: (ii) −*x*+1, *y*−1/2, −*z*+3/2.
